# Supplementary material for: Molecular Manipulation of the miR160/AUXIN RESPONSE FACTOR Expression Module Impacts Root Development in Arabidopsis thaliana
Source: Genes (Basel). 2024 Aug 7;15(8):1042. doi: 10.3390/genes15081042 (PMC11353855; doi:10.3390/genes15081042)
Supplement: Supplementary file 1 [file genes-15-01042-s001.zip › genes-3119449-supplementary.pdf]

Table S1. DNA oligonucleotides used to construct the *MIR160B*, *mARF10* and *mARF16* transgenes.

Name	*Target*	*Sequence (5′ to 3′)*
pARF10-Pro-F	*ARF10* promoter	TCACTCGAGAATGCAAGACAACCCACCAA
pARF10-Pro-R		TCAGGATCCCTAGACGAAGTTGTGTAACC
pARF16-Pro-F	*ARF16* promoter	TCACTCGAGTGGATTCATCACATTTATCAT
pARF16-Pro-R		TCATCTAGAACATGCGGAAATTTATTGAGC
pMIR160B-OE-F	*MIR160B* precursor	TCACTCGAGGCACGCTGTGTCTGTCTCTTT
pMIR160B-OE-R		TCAGGATCCTGCCTTGATTGGAAGATCTGA

Table S2. DNA oligonucleotides used in this study for cDNA synthesis or the analysis of gene expression.

Name	Target	Sequence (5′ to 3′)
Stem-Loop RT-qPCR Primers		
pSNO101-F	sno101	CTTCACAGGTAAGTTCGCTTG
pSNO101-R		AGCATCAGCAGACCAGTAGTT
pmiR160-cDNA-R	miR160	GTCGTATCCAGTGCAGGGTCCGAGGTATTCGCACTGGATACGACTGGCAT
pmiR160-cDNA-F		GCTGCCTGGCTCCCTGT
pGeneric-STL		CCAGTGCAGGGTCCGAGGTA
RT-PCR Primers		
pACT2-RTF	*ACTIN2*	TCTTCCGTCTTTCTTTCCA
pACT2-RTR		GAGAGAACAGCTTGGATGGC
pARF10-RTF	*ARF10*	CGGTACTAAATTCCCGATTTTCT
pARF10-RTR		GAATGTAACTTGTTGTTACCGGTGT
pARF16-RTF	*ARF16*	CAGTACCTTCATTCCCAAGCA
pARF16-RTR		GATGTTTCGGAGACCGAGAG
pARF17-RTF	*ARF17*	AGCAGCACCTGATCCAAGTC
pARF17-RTR		GTCGACACTTTTCCCAAATCA
RT-qPCR Primers		
pMIR160A-F	*PRE-MIR160A*	ATATGCTGAGCCCATCGAGTATCG
pMIR160A-R		ATGCATGGCTCCTCATACGCC
pMIR160B-F	*PRE-MIR160B*	GCCACAAGAAAACATCGATTTAGTTTC
pMIR160B-R		TGCTTGACTACTCTGTACGCCA
pMIR160C-F	*PRE-MIR160C*	CCACGAGTGGATACCGATTTTG
pMIR160C-R		GCTTGACTCCTTGTACGCCAC
pETM160-1F	*eTM160-1*	TCTTCAGAGATGGCCTGACGA
pETM160-1R		AATCGTAATCCTAATCAGTGTT
pETM160-2F	*eTM160-2*	ACCGGACTGTCAGTGCTTGAT
pETM160-2R		TTCGCAAATGTCACTCCAAAA
pARF10-F	*ARF10*	CGGTTTTTGGAAGAAGAGGCGG
pARF10-R		GCGTCCAACATCCTCAGATTCCAT
pARF16-F	*ARF16*	AACTTTCTCCTTCTCTCGGTCTCCG
pARF16-R		AGCTTGCCGAACAATACAATATGGG
pARF17-F	*ARF17*	CGAGTCAAGATGGCTATGGA
pARF17-R		CATCCCATGTGATCTGAAGC
pDRB1-F	*DRB1*	ATGACCTCCACTGATGTTTCC
pDRB1-R		TGCTAATTCCCGGAGAGC
pDRB2-F	*DRB2*	ATGTATAAGAACCAGCTACAAGAGTTG
pDRB2-R		CAGCAGCAGAGTGTTCAGC
pDRB4-F	*DRB4*	AAATGGGAACTCGAACCAGA
pDRB4-R		CCACCTTGGAAGAAGGTTGA
pEF1-A-F	*EF1-*α	TGAGCACGCTCTTCTTGCTTTCA
pEF1-A-R		GGTGGTGGCATCCATCTTGTTACA
PCR-based Genotyping Primers		
pORE1-GENO-F	pORE1	GCTGATATGGCCGCTGTTTGT
pORE1-GENO-R		CAATGTACCCCTGGCTGTGT
pBART-GENO-F	pBART	CATCGAGACAAGCACGGTCA
pBART-GENO-R		AAACCCACGTCATGCCAGTT

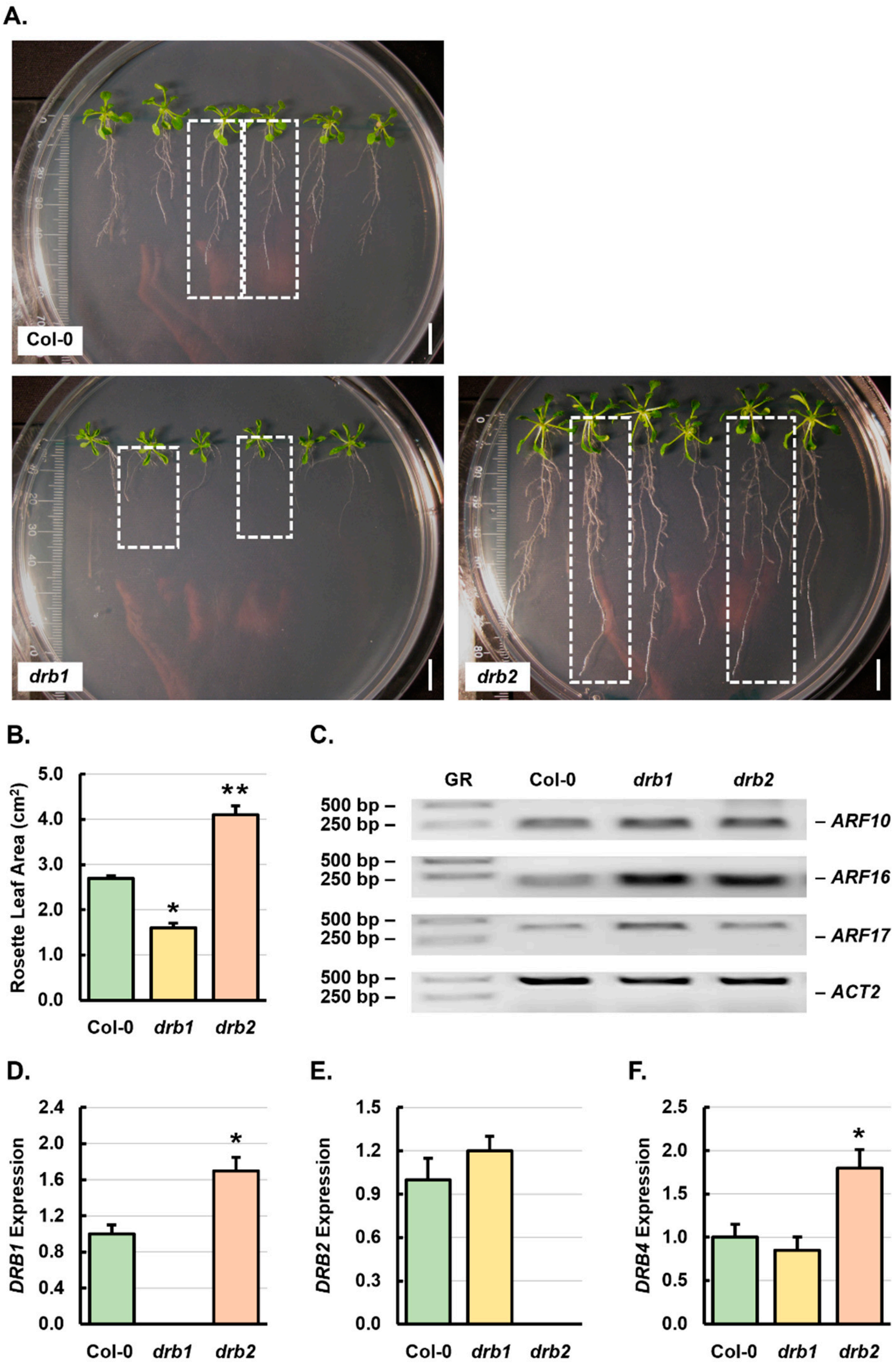

Figure S1. Assessment of the miR160/*ARF10*/*ARF16*/*ARF17* expression module in wild-type *Arabidopsis* plants and the *drb1* and *drb2* single mutants. (**A**) Whole plant phenotypes expressed by 3-week-old Col-0 plants and the *drb1* and *drb2* single mutants. Scale bars = 1.0 cm. (**B**) Rosette leaf area (cm^2) of 3-week-old Col-0, *drb1* and *drb2* plants. (**B**) Semi-quantitative RT-PCR analysis of *ARF10*, *ARF16* and *ARF17* expression in the inflorescences of Col-0, *drb1* and *drb2* plants. The *ACTIN2* (*ACT2*; *AT3G18780*) gene was used as the loading control for this analysis. (**D–F**) RT-qPCR assessment of *DRB1* (**D**), *DRB2* (**E**) and *DRB4* (**F**) expression in Col-0, *drb1* and *drb2* inflorescences. Fold changes were determined by the ΔΔCt method with the use of three biological replicates. Averages of expression are represented as a fold change for each assessed transcript and were compared to the values obtained for Col-0 plants by a standard two-tailed *t*-test. Error bars represent the standard error of the mean (SEM) and asterisks show * $p \leq 0.05$, ** $p \leq 0.01$, *** $p \leq 0.001$.

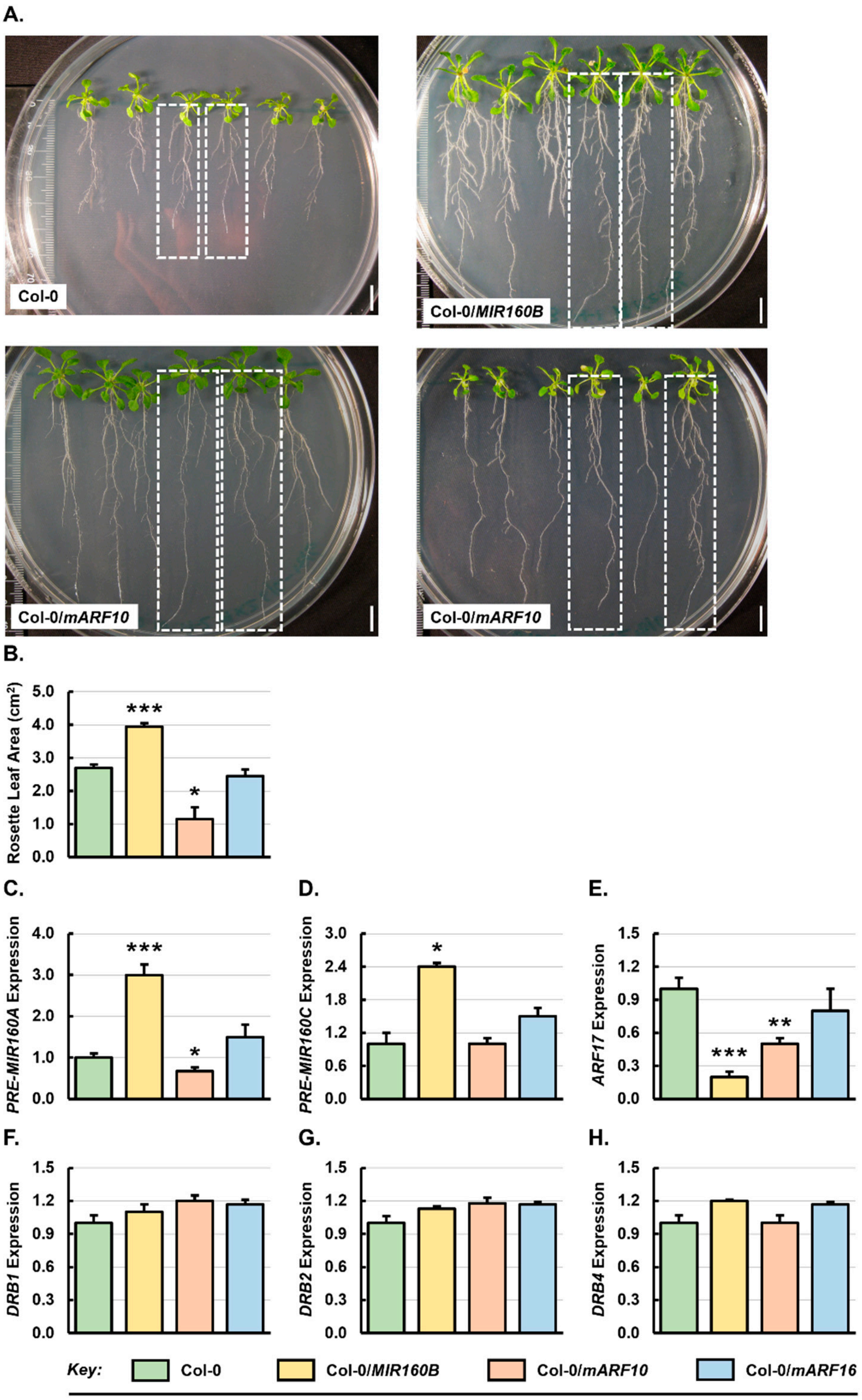

Figure S2. Phenotype displayed by and molecular assessment of Col-0, Col-0/*MIR160B*, Col-0/*ARF10* and Col-0/*ARF16* plants. (**A**) Representative whole plant images of 3-week-old Col-0, Col-0/*MIR160B*, Col-0/*ARF10* and Col-0/*ARF16* plants. Scale bars = 1.0 cm. (**B**) Rosette leaf area (cm^2) of 3-week-old Col-0, Col-0/*MIR160B*, Col-0/*ARF10* and Col-0/*ARF16* plants. (**C–H**) RT-qPCR assessment of *PRE-MIR160A* (**C**), *PRE-MIR160C* (**D**), *ARF17* (**E**), *DRB1* (**F**), *DRB2* (**G**) and *DRB4* (**H**) in the roots of 3-week-old Col-0, Col-0/*MIR160B*, Col-0/*ARF10* and Col-0/*ARF16* plants. Fold changes were determined by the ΔΔCt method with the use of three biological replicates. Averages of expression are represented as a fold change for each assessed transcript and were compared to the values obtained for Col-0 plants by a standard two-tailed *t*-test. Error bars represent the standard error of the mean (SEM) and asterisks show * $p \leq 0.05$, ** $p \leq 0.01$, *** $p \leq 0.001$.

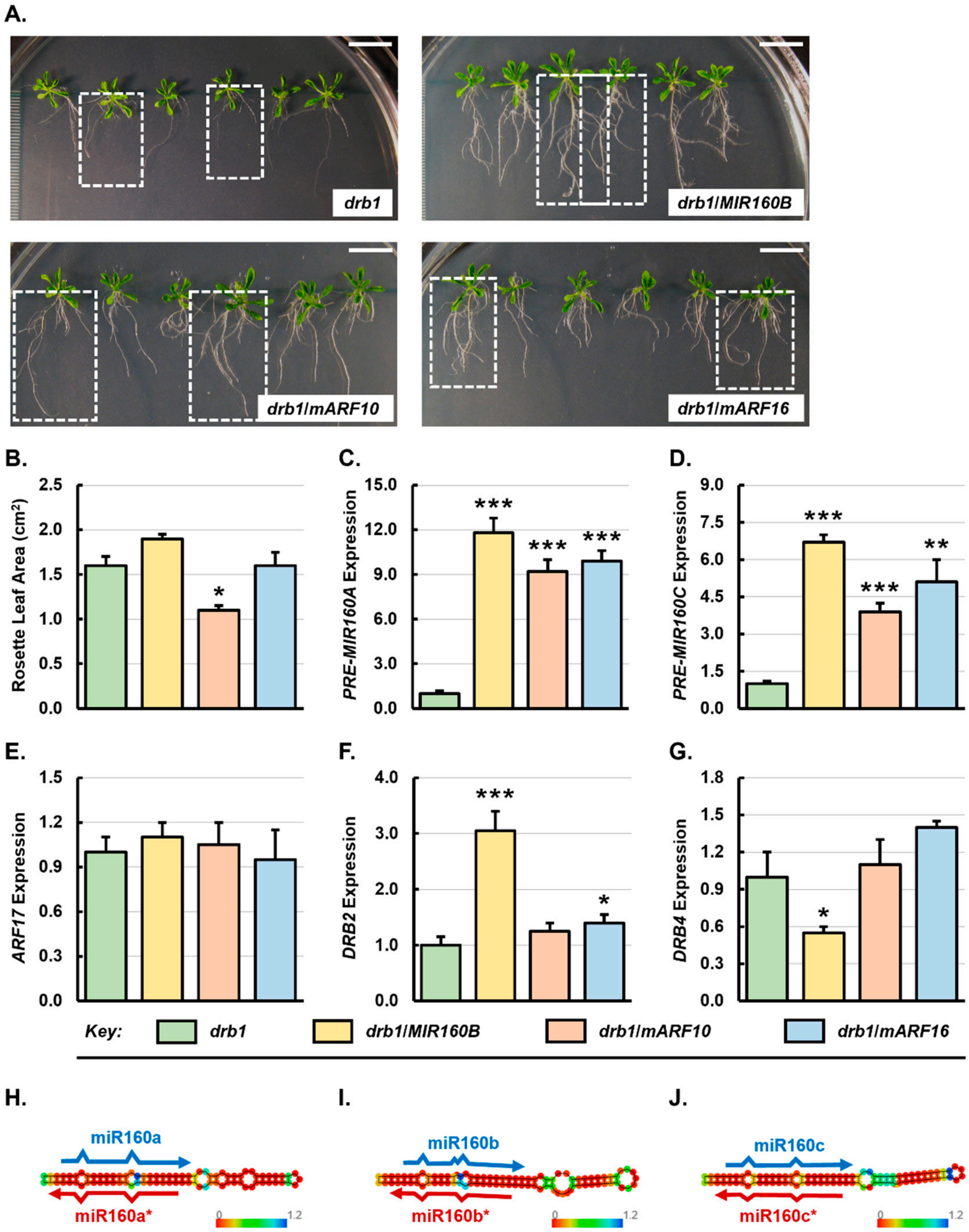

Figure S3. Phenotype displayed by and molecular assessment of *drb1*, *drb1/MIR160B*, *drb1/ARF10* and *drb1/ARF16* plants. (**A**) Representative whole plant images of 3-week-old *drb1*, *drb1/MIR160B*, *drb1/ARF10* and *drb1/ARF16* plants. Scale bars = 1.0 cm. (**B**) Rosette leaf area (cm^2) of 3-week-old *drb1*, *drb1/MIR160B*, *drb1/ARF10* and *drb1/ARF16* plants. (**C–G**) RT-qPCR assessment of *PRE-MIR160A* (**C**), *PRE-MIR160C* (**D**), *ARF17* (**E**), *DRB2* (**F**), and *DRB4* (**G**) expression in the roots of 3-week-old *drb1*, *drb1/MIR160B*, *drb1/ARF10* and *drb1/ARF16* plants. Fold changes were determined by the ΔΔCt method with the use of three biological replicates. Averages of expression are represented as a fold change for each assessed transcript and were compared to the values obtained for *drb1* plants by a standard two-tailed *t*-test. Error bars represent the standard error of the mean (SEM) and asterisks show * $p \leq 0.05$, ** $p \leq 0.01$, *** $p \leq 0.001$. (**H–J**) RNAfold Web Server (http://rna.tbi.univie.ac.at/cgibin/RNAWebSuite/RNAfold.cgi) generated schematics depicting the folding structures of the *PRE-MIR160A*, *PRE-MIR160B* and *PRE-MIR160C* transcripts, the three precursors that the miR160 sRNA is liberated from in *Arabidopsis*.

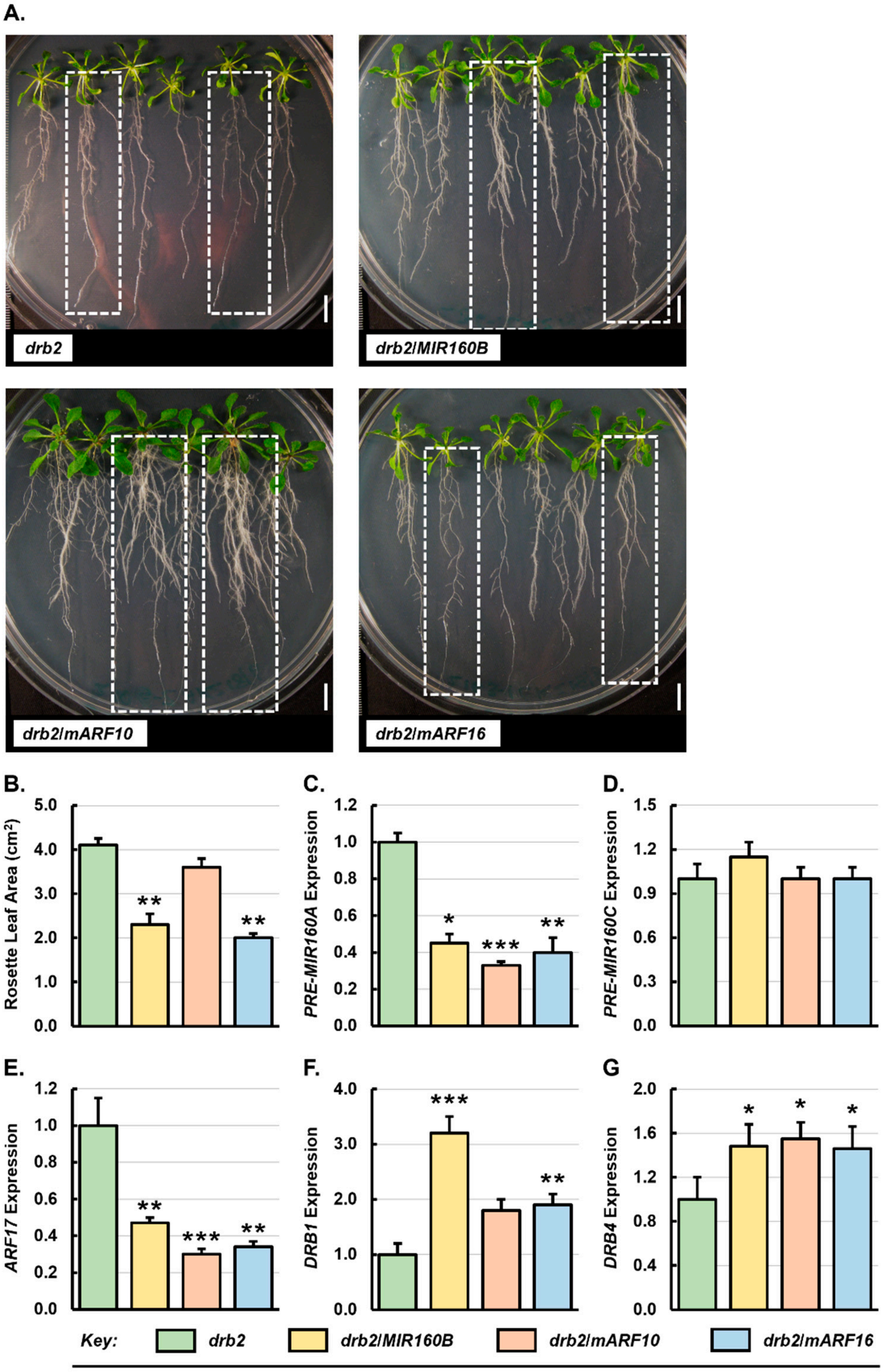

Figure S4. Phenotype displayed by and molecular assessment of *drb2*, *drb2/MIR160B*, *drb2/ARF10* and *drb2/ARF16* plants. (**A**) Representative whole plant images of 3-week-old *drb2*, *drb2/MIR160B*, *drb2/ARF10* and *drb2/ARF16* plants. Scale bars = 1.0 cm. (**B**) Rosette leaf area (cm^2) of 3-week-old *drb2*, *drb2/MIR160B*, *drb2/ARF10* and *drb2/ARF16* plants. RT-qPCR assessment of *PRE-MIR160A* (**C**), *PRE-MIR160C* (**D**), *ARF17* (**E**), *DRB1* (**F**), and *DRB4* (**G**) expression in the roots of 3-week-old *drb2*, *drb2/MIR160B*, *drb2/ARF10* and *drb2/ARF16* plants. Fold changes were determined by the ΔΔCt method with the use of three biological replicates. Averages of expression are represented as a fold change for each assessed transcript and were compared to the values obtained for *drb2* plants by a standard two-tailed *t*-test. Error bars represent the standard error of the mean (SEM) and asterisks show * $p \leq 0.05$, ** $p \leq 0.01$, *** $p \leq 0.001$.
